# Effect of Compatibilizer on the Interface Bonding of Graphene Oxide/Polypropylene Composite Fibers

**DOI:** 10.3390/polym10111283

**Published:** 2018-11-18

**Authors:** Miao Miao, Chunyan Wei, Ying Wang, Yongfang Qian

**Affiliations:** Department of Textile and Material Engineering, Dalian Polytechnic University, Dalian 116034, China; m1a0_1993@163.com (M.M.); wangying@dlpu.edu.cn (Y.W.); qianyf@dlpu.edu.cn (Y.Q.)

**Keywords:** graphene oxide, polymer composite fiber, interfacial bonding, polypropylene, thermal stability

## Abstract

To improve the interfacial bonding and thermal stability of graphene oxide (GO)/polypropylene (PP) composite fibers, a composite fiber with PP as the matrix, GO as reinforcement and maleic anhydride-grafted PP (PP-g-MAH) as a compatibilizer was prepared by a simple and efficient melt-blending method. The GO content was 0.0–5.0 wt %. According to the Fourier Transform Infrared (FT-IR) spectroscopy results, the interfacial bonding in the PP/MAH/GO composite fibers was improved. The Dynamic Mechanical Analysis (DMA) results show that the addition of GO resulted in better interfacial adhesion and higher storage modulus (*E*′). The loss modulus (*E*″) of the PP/MAH/GO-*x* composite fibers increased with increasing amount of added GO, whereas the loss factor (tan δ) decreased. GO and PP-g-MAH were analyzed by Thermogravimetric Analysis (TGA). The thermal stability of the composite fibers was improved compared to PP. Differential Scanning Calorimetry (DSC) analysis showed that the addition of PP-g-MAH to the composite fiber improved the interfacial bonding of GO in the PP matrix. Thus, compatibility between the two components was obtained. Based on the Scanning Electron Microscopy (SEM) results, the PP fibers exhibited relative orientation due to the strong crystalline morphology. The rough section, PP/GO blend fiber exhibits a very clear phase separation morphology due to the incompatibility between the two and the compatibility of GO and PP in PP/MAH/GO-3 composite fiber is improved, resulting in the interface between the two has improved.

## 1. Introduction

Polypropylene (PP), a thermoplastic polyolefin, has excellent characteristics such as low cost, high strength, good dimensional stability, friction resistance and chemical resistance. PP is widely used in textile fibers, packaging and labelling and automobiles parts, industries, agriculture and other fields have been widely concerned in recent years [1]. However, the non-polar chain structure of PP makes it difficult to combine with reinforcing materials [2]. Nowadays, this is a problem which should be solved. Therefore, considerable research has been done on the development of materials with modified PP. The modification of PP can be done using the ultraviolet light irradiation grafting method [3,4], plasma graft modification method [5,6] and melt-blending method [7]. The melt blending of polymers is a convenient and effective method for designing new high-performance polymers [8]. Blends of two or more polymers can balance performance, highlighting the respective advantages of the blends to mask the disadvantages. However, due to their different chemical structures and polarities, most polymer blends are thermodynamically immiscible and exhibit poor mechanical properties, poor interfacial adhesion and unstable morphology [9]. Therefore, we used maleic anhydride-grafted PP (PP-g-MAH) as a compatibilizer. The MAH group on the PP-g-MAH segment helps to improve the compatibility between graphene oxide (GO) and PP [10,11], thereby improving the interfacial bonding between GO and PP.

In recent years, inorganic nanomaterials have provided new modification methods for incompatible blends, such as carbon black [12], nanoclay [13], carbon nanotubes [14], graphene and its derivatives [15] and glass fibers [16]. Among inorganic nanomaterials, GO has a typical quasi-two-dimensional structure [17] and its layer contains many oxygen-containing groups. Because of its high specific surface energy, good hydrophilicity and good mechanical properties [18], GO is considered as an ideal polymer nano-based composite fiber additive for polymers. Tiwari et al. investigated the effects of different GO loadings on the compatibility, thermomechanical and morphological properties of incompatible PP/polycarbonate polymer blends [19]. Botlhoko et al. comparatively investigated the effects of graphite (G) and GO dispersions on the thermal, mechanical and rheological properties of biodegradable polylactide (PLA)/poly(07-caprolactone) (PCL) blends [20]. Wang et al. employed GO as a two-dimensional nanofiller and nucleating agent to improve the properties of immiscible PLA/PCL blends with weight ratios of 70/30, 50/50 and 30/70 [21]. Chen et al. prepared polyamide-6/graphene-graphene oxide composites with super-high thermal conductivity through in situ polymerization and the interface adhesion enhanced by adding small amounts of graphene-GO [22]. Meng et al. prepared polyamide-6/graphite nanoflakes by situ intercalation polymerization approach for enhanced thermal conductivity [23].

In this study, PP was used as the matrix, GO was used as a reinforcement and PP-g-MAH was used as a compatibilizer. Composites containing GO contents of 0.0–5.0 wt % were prepared using a melt-blending method. The interfacial bonding properties, thermal properties, mechanical properties and morphological characteristics of the composite fibers were studied with GO content as the independent variable.

## 2. Experiment

### 2.1. Materials

Polypropylene (melt flow rate 8.0 g/10 min) was purchased from Sinopec Hainan petrochemical Co., Ltd. (Yangpu Economic Development Zone, Hainan, China); Maleic anhydride grafted polypropylene (MA content of 1.3%, melt flow rate of 150 g/10 min at 230 °C and 2.16 kg) was supplied from Hebei Xintianqi Plastic Co., Ltd., (Hebei, China); Multilayer graphene oxide (thickness 3.4–7.0 nm, sheet diameter 10–50 μm)was purchased from Suzhou Tanfeng Technology Co., Ltd., Suzhou, China.

### 2.2. Preparation of Composite Fiber

**Figure 1:**
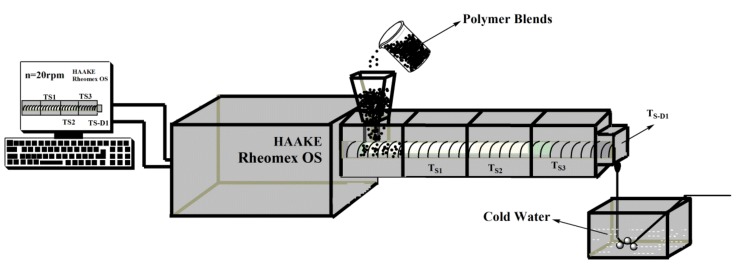
Schematic diagram of the melt spinning device.

PP and PP-g-MAH were first mixed in proportion and the mixture was melt-compounded and extruded with different contents of GO using a Huck single-screw extruder (HAAKE Polylab OS, Karlsruhe, Germany). This experiment used the single hole spinneret with a diameter of 1.5 mm. To effectively mix the mixture, the temperature of the single-screw extruder from the barrel to the head was set to 150, 180, 200 and 200 °C. The screw speed was fixed at 20 rpm. A schematic diagram of the melt spinning device is shown in Figure 1. The extruded mixture was cooled by cold water and dried in an oven for 24 h for subsequent testing and analysis. The GO content in PP/PP-g-MAH (3/1) ranged from 0.0 to 5.0 wt %. The composite fibers were named PP/MAH/GO-*x*, where *x* represents the GO weight percentage.

### 2.3. Characterization

The molecular structure of the composite fiber was analyzed by Fourier Transform Infrared spectroscopy (FT-IR, Waltham, MA, USA). The sample to be tested and the potassium bromide powder were thoroughly ground and mixed in an agate mortar at a mass ratio of 1:100. Press into the mold and press into a sheet on the press. The spectral range is 4000–500 cm^−1^.

The dynamic storage modulus and loss tangent angle of the composite fiber were measured by a Dynamic Mechanical Analyzer (DMA-Q800, TA, New Castle, DE, USA). The sample clamping length was 10.5 ± 0.5 mm and the diameter was 1.3 ± 0.1 mm. In the tension mode, the temperature range was −50 °C–150 °C. The heating rate was 5 °C/min and the frequency was 1 Hz.

The thermal stability of the composite fibers was tested using a Thermogravimetric Analyzer (TGA, SDT-Q600, TA, New Castle, DE, USA). About 10 mg of the sample was placed in the SDT-Q600 at a heating rate of 10 °C/min, rising from room temperature to 550 °C.

The Differential Scanning Calorimeter (DSC-Q2000, TA, New Castle, DE, USA) was used to identify the thermal transition behavior of the composite fiber. About 10mg of the sample was placed in the DSC-Q2000 at a heating rate of 10 °C/min, rising from −50 °C to 200 °C, naturally cooling to 30 °C, eliminating the heat history and then, the glass transition temperature (*Tg*) of the sample was tested under a nitrogen atmosphere at a temperature increase rate of 10 °C/min from −50 °C to 200 °C.

The cross-sectional morphology of the composite fibers was characterized by Scanning Electron Microscopy (SEM, JSM-7800F, JEOL, Tokyo, Japan). The fibers were quenched at low temperature in liquid nitrogen and their cross sections were observed.

## 3. Results and Analysis

### 3.1. FT-IR Analysis of Composite Fibers

**Figure 2:**
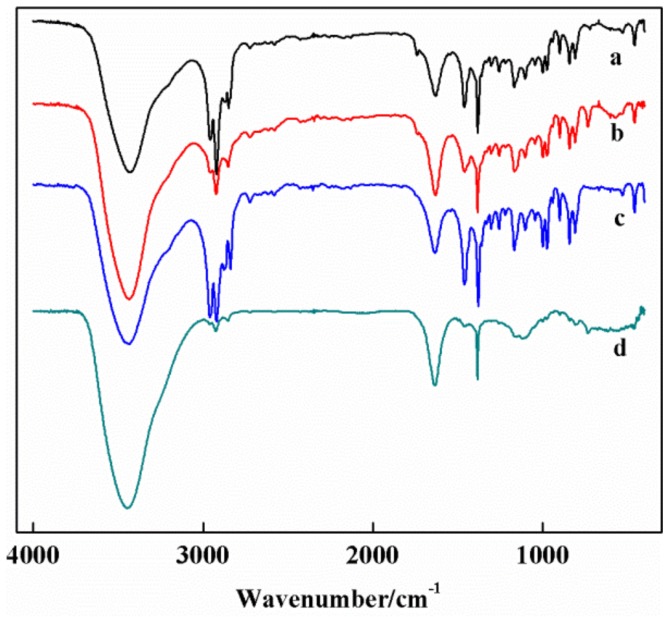
FT-IR analysis chart (**a**) PP; (**b**) GO; (**c**) PP/GO blend fiber; (**d**) PP/MAH/GO-3 composite fiber).

**Figure 3:**
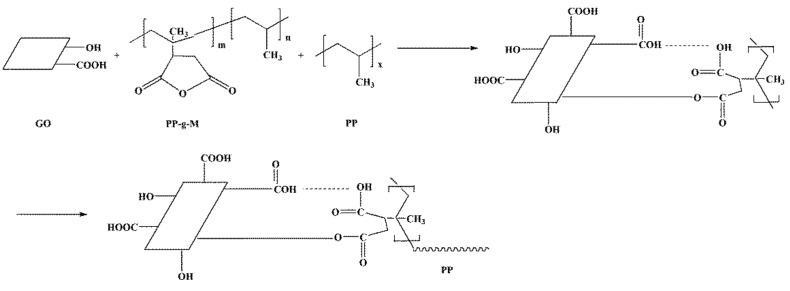
Composite fiber reaction mechanism diagram.

Figure 2 shows the infrared spectra of pure PP, GO, PP/GO blend fibers and PP/MAH/GO-3 composite fibers. The absorption band of PP (Figure 2a) has four main characteristic peaks and the characteristic absorption peaks at 2951 and 2872 cm^−1^ correspond to the asymmetric and symmetric stretching of the methyl group. The characteristic absorption peaks at 2920 and 2850 cm^−1^ are attributed to the asymmetric and symmetric stretching of the methylene groups. The characteristic absorption peak in the spectrum of GO (Figure 2b) at 1727 cm^−1^ corresponds to the stretching of C=O. The characteristic absorption peaks at 1621 and 1384 cm^−1^ are attributed to the stretching of C=C and –OH, respectively. The hydroxyl group in GO acts as a reactive group that chemically interacts with the MAH group to improve the interfacial bonding between GO and PP. The infrared spectrum of the PP/MAH/GO composite fiber shows new absorption peaks at 2923 and 2854 cm^−1^, corresponding to the stretching of –CH_2_ on the PP-g-MAH molecular chain. The characteristic peak at 2951 cm^−1^ is attributed to –CH_3_ stretching. When two polymer materials are blended, if simple physical blending alone does not produce intermolecular forces, the FT-IR spectrum of the blend will simply be a superposition of the spectra of the two components, as for the PP/GO fibers (Figure 2c). If intermolecular interactions (e.g., hydrogen bonds) occur between the two components of the blend, a new spectrum that is not simply the superposition of peaks will be generated, as for the PP/MAH/GO composite fibers. The generation of hydrogen bonds causes some absorption peaks to shift in position and shape. The anhydride group of MAH can be esterified with the macromolecular alcohol hydroxyl group under melt-blending conditions and the intermolecular reaction improves the interfacial compatibility between the blend components to some extent. After adding MAH, the PP/MAH/GO-3 (Figure 2d) composite fibers and pure PP fibers showed similar peaks. However, it belongs to the vibration absorption peak of the ester-based band (at 1127 cm^−1^) and the stretching vibration peak of C=C (at 1621 cm^−1^) and the intensity increases remarkably. This indicates that in the experimental range, in addition to the physical state mixing of GO and PP-g-MAH molecular chains in the PP/MAH/GO composite fibers, a certain degree of esterification occurred. The reaction mechanism is shown in Figure 3. The esterification reaction promotes the compatibility between the molecular chains, thereby improving interfacial bonding in the PP/MAH/GO composite fibers.

### 3.2. Dynamic Mechanical Properties of Composite Fibers

**Figure 4:**
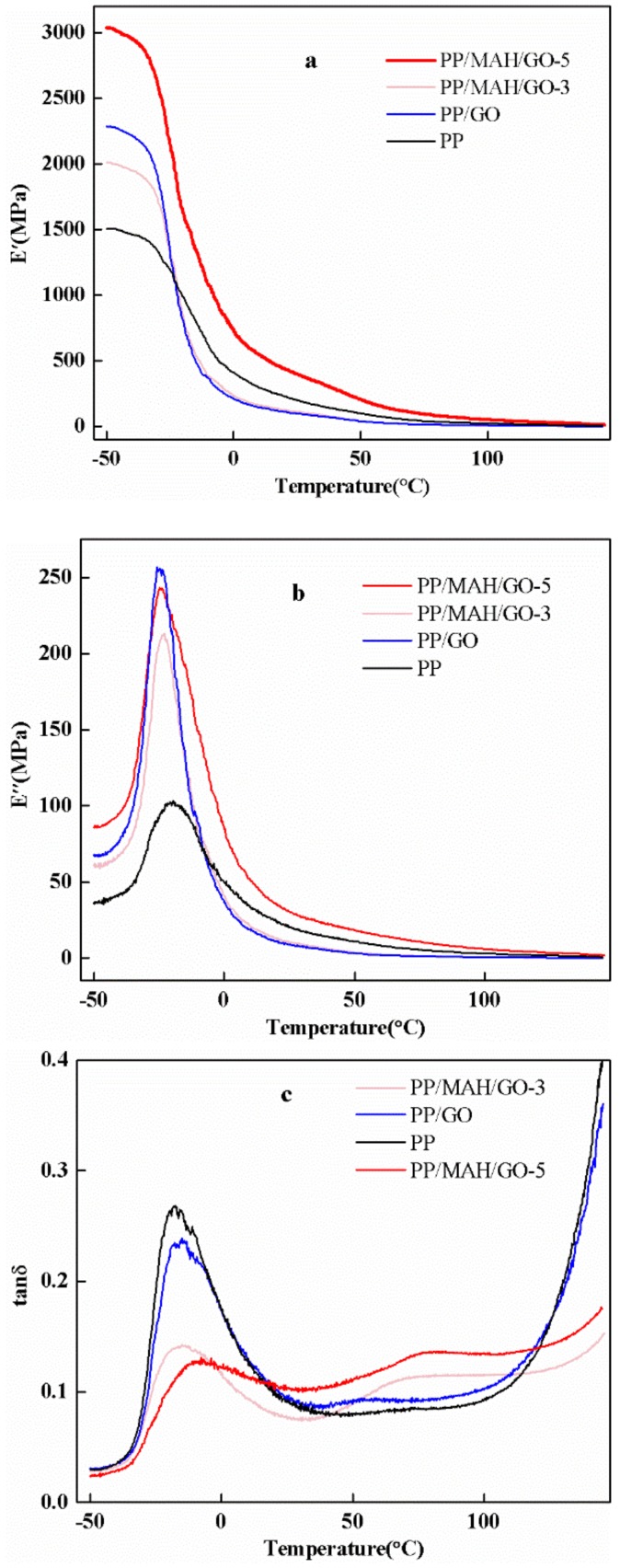
DMA image of pure PP and composite fibers: (**a**) *E*′; (**b**) *E*″; (**c**) tan δ.

DMA is an effective method for evaluating the interfacial interactions in reinforced composite fibers. To characterize the mechanical properties of composite fibers with different GO weight fractions as a function of temperature, the dynamic mechanical data, storage modulus (*E*′), loss modulus (*E*″) and loss factor (tan δ) were evaluated (Figure 4).

Figure 4a shows the E′ values of pure PP fibers, PP/GO blended fibers and PP/MAH/GO-*x* composite fibers as functions of temperature. *E*′ is similar to the bending modulus (*E*), which describes the stiffness of the material. Compared to pure PP, the storage modulus temperature of PP/GO blend fibers and the change are similar to those of pure PP fibers. However, the *E*′ values of the blended fibers are higher than that of pure PP because of the presence of GO. The high *E*′ value of the PP/MAH/GO-*x* composite fibers indicates good interfacial adhesion.

Figure 4b shows *E*″, which generally indicates the viscosity of the material, as a function of time for the pure PP fibers, PP/GO blended fibers and PP/MAH/GO-*x* composite fibers. Similar to *E*′, *E*″ also increased upon the addition of GO. This phenomenon is attributed to the interactions between the fibers and matrix, which limit the movement of PP molecules, resulting in a higher viscosity and increased *E*″. On the other hand, if the fibers interact very strongly with the matrix, *E*″ will decrease because of the absence of fiber slip and energy dissipation. This is why *E*″ of PP/MAH/GO-5 is lower than *E*″ of PP/MAH/GO-3. This conclusion is consistent with Wei et al. [2].

In general, the peak temperature of the loss factor corresponds to the glass transition temperature (*Tg*) of a material. In a binary blend without a compatibilizer, the loss factor has two peaks in the curve over time. These peaks correspond to the *Tg* values of both materials. After the addition of compatibilizer, the dispersibility of the dispersed phase is improved, the interaction between interfaces is enhanced, the degree of bonding between particles is improved and the compatibility of the system is remarkably improved. However, a homogeneous blended system is equivalent to a random copolymer. Thus, there is only one *Tg* value and only one peak in the loss factor curve. As the compatibility of the blend improves, the *Tg* values of the two phases in the blend will interact with each other. Close, there is a tendency to form a *Tg* [2]. Figure 4c shows plots of tan δ for pure PP, PP/GO blended fibers and PP/MAH/GO composite fibers. The loss factor of the PP/MAH/GO composite fibers showed only one peak, which corresponded to the *Tg* of PP. This indicates that the compatibility between PP and GO increased after adding PP-g-MAH, which is consistent with the FT-IR results. Moreover, the tan δ values of all composite fibers are lower than those of pure PP fibers, which is attributed to the strengthening of GO and the limitation of polymer molecular motion. As shown in Table 1, the maximum loss factor decreased as the amount of added GO increased.

### 3.3. TGA of Composite Fibers

**Figure 5:**
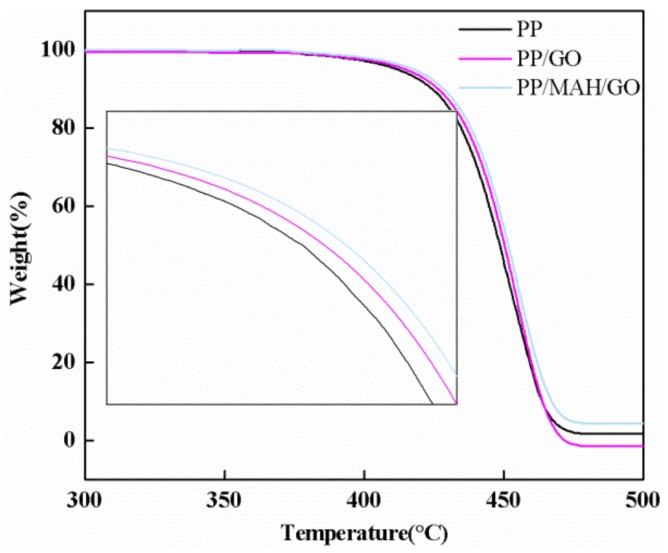
TGA image of composite fibers.

To analyze the degradation temperature and thermal stability of the composite fibers, TGA was conducted. Figure 5 shows weight losses of pure PP and composite fibers with temperature. The thermal degradation of pure PP mainly occurred in the range of 410 °C to 470 °C. With the addition of PP-g-MAH, the initial degradation temperature of the blend shifted into the high-temperature zone, indicating that the addition of PP-g-MAH increased the thermal stability of the composite fibers. This phenomenon is explained as follows. On one hand, PP-g-MAH itself has a certain degree of crystallinity and heat resistance and is capable of absorbing a certain amount of heat and storing it in the form of heat energy. On the other hand, the interaction between GO and PP-g-MAH causes the concentration of the entanglement point to increase, which increases the outward diffusion of thermal energy to a certain extent and hinders alkyl oxygen cleavage. Thus, the thermal stability of the composite fibers is improved with respect to PP. These results are consistent with the FT-IR spectra.

### 3.4. Thermal Transition Behavior Analysis of Composite Fibers

**Figure 6:**
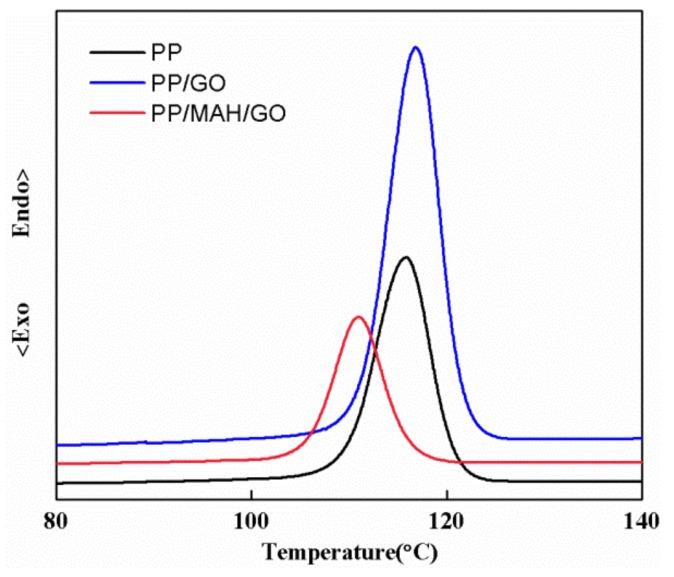
Crystallization temperature curve of PP, PP/GO and PP/PP-g-MAH/GO composite fibers.

The crystallization temperature (*Tc*) of the PP fibers, PP/GO fibers and PP/PP-g-MAH/GO composite fibers were determined by DSC (Figure 6). The *Tc* peak is usually used to indicate the crystal structure. The *Tc* of the PP fibers was 115.8 °C and a semi-crystalline state formed as a result of the rapid crystallization rate of PP. No glass transition pattern or cold crystallization temperature was detected in the PP fibers. After adding GO, the *Tc* of the composite fibers increased, which is attributed to an increase in GO nucleation ability. Adding PP-g-MAH to the composite fiber improved the interfacial bonding of GO in the PP matrix along with the compatibility between the reinforcement and matrix and it is difficult to crystallize. Therefore, the *Tc* of the PP/PP-g-MAH/GO composite fibers shifted towards lower temperature.

### 3.5. SEM of Composite Fibers

**Figure 7:**
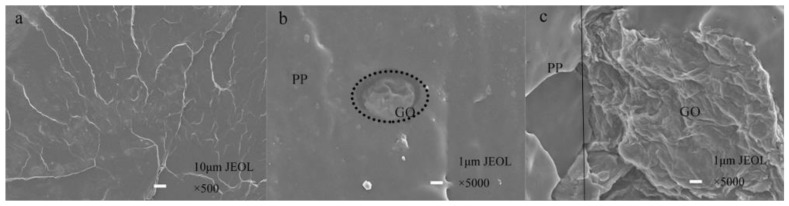
SEM image of low temperature fracture interface of PP, PP/GO blended fiber and PP/PP/GO composite fiber (**a**) pure PP fiber; (**b**) PP/GO blend fiber; (**c**) PP/MAH/GO-3 composite fiber).

To detect the effect of interfacial bonding between GO and the PP matrix, the images of PP fibers, PP/GO blended fibers and PP/MAH/GO composite fibers at low temperature fracture interface were obtained by SEM (Figure 7). Figure 7a shows a cross-sectional view of pure PP fibers under liquid nitrogen, revealing a relatively rough cross section attributed to the strong crystalline morphology of PP. Figure 7b shows a cross-sectional SEM image of a PP/GO blended fiber. As a result of the incompatibility between PP and GO, the interface in the PP/GO blended fiber is clear and a distinct phase separation is apparent. Figure 7c shows a cross-sectional SEM image of a PP/MAH/GO-3 composite fiber. The compatibility between GO and PP in the composite fiber was improved with respect to the PP/GO fiber, in agreement with the FT-IR and DMA results.

## 4. Conclusions and Prospects

In this study, PP was used as the matrix, GO was the reinforcement and PP-g-MAH was the compatibilizer. Composite fibers with GO contents of 0.0–5.0 wt % were prepared by a simple and efficient melt-blending method. FT-IR, DMA and DSC showed that the compatibility between GO and PP along with the interfacial compatibility in the PP/MAH/GO composite fibers were improved compared to in the PP/GO blended fibers. TGA demonstrated that the thermal stability of the composite fibers was higher than that of PP. SEM analysis verified that the PP fibers showed relatively rough cross sections resulting from the strong crystalline morphology of PP. The PP/GO blended fibers showed clear phase separation resulting from the incompatibility between the two phases. Compared to the PP/GO blended fibers, the compatibility between GO and PP was much improved in the PP/MAH/GO-3 composite fibers, resulting in enhanced interfacial bonding between GO and PP. The current study is only the first step in studying the performance of PP nonwoven materials. In the future, composite fibers will be used for the preparation of PP nonwoven fabrics and their antistatic properties, aging resistance, electrical conductivity and comfort performance will be evaluated.

## Figures and Tables

**Table 1:** The DMA analysis results of pure PP and composite fibers.

Sample Name	*E*′(MPa)	*E*″(MPa)	$\tan\delta_{\max}$	*Tg* (°C)
PP	2913	253	0.296	−17.91
PP/GO	2188	241	0.235	−16.56
PP/MAH/GO-0.3	1923	212	0.14	−16.15
PP/MAH/GO-0.5	1439	99.8	0.12	−10.61
